# Leveraging a disulfidptosis-related signature to predict the prognosis and immunotherapy effectiveness of cutaneous melanoma based on machine learning

**DOI:** 10.1186/s10020-023-00739-x

**Published:** 2023-10-26

**Authors:** Yi Zhao, Yanjun Wei, Lingjia Fan, Yuanliu Nie, Jianan Li, Renya Zeng, Jixian Li, Xiang Zhan, Lingli Lei, Zhichao Kang, Jiaxin Li, Wentao Zhang, Zhe Yang

**Affiliations:** 1 Tumor Research and Therapy Center, Shandong Provincial Hospital Affiliated to Shandong First Medical University, Jinan, Shandong China; 2 Department of Radiation Oncology, Weifang People’s Hospital, Weifang, China; 3 Department of Orthopaedic Surgery, Taian Central Hospital, Taian, Shandong China

**Keywords:** Disulfidptosis, Immune, Cutaneous melanoma, Prognosis, Signature

## Abstract

**Background:**

Disulfidptosis is a recently discovered programmed cell death pathway. However, the exact molecular mechanism of disulfidptosis in cutaneous melanoma remains unclear.

**Methods:**

In this study, clustering analysis was performed using data from public databases to construct a prognostic model, which was subsequently externally validated. The biological functions of the model genes were then investigated through various experimental techniques, including qRT-PCR, Western blotting, CCK-8 assay, wound healing assay, and Transwell assay.

**Results:**

We constructed a signature using cutaneous melanoma (CM) data, which accurately predicts the overall survival (OS) of patients. The predictive value of this signature for prognosis and immune therapy response was validated using multiple external datasets. High-risk CM subgroups may exhibit decreased survival rates, alterations in the tumor microenvironment (TME), and increased tumor mutation burden. We initially verified the expression levels of five optimum disulfidptosis-related genes (ODRGs) in normal tissues and CM. The expression levels of these genes were further confirmed in HaCaT cells and three melanoma cell lines using qPCR and protein blotting analysis. HLA-DQA1 emerged as the gene with the highest regression coefficient in our risk model, highlighting its role in CM. Mechanistically, HLA-DQA1 demonstrated the ability to suppress CM cell growth, proliferation, and migration.

**Conclusion:**

In this study, a novel signature related to disulfidptosis was constructed, which accurately predicts the survival rate and treatment sensitivity of CM patients. Additionally, HLA-DQA1 is expected to be a feasible therapeutic target for effective clinical treatment of CM.

**Supplementary Information:**

The online version contains supplementary material available at 10.1186/s10020-023-00739-x.

## Introduction

Cutaneous melanoma arises from the malignant transformation of melanocytes. Despite representing only about 1% of skin cancer cases, it is the most lethal form (Guo et al. 2021). CM incidence is influenced by race, lifestyle, and genetics, with acral and mucosal subtypes being predominant in Asian populations (Mao et al. 2021). The high degree of immunogenicity in CM has rendered immunotherapy an indispensable component in its treatment. However, despite the advancements in targeted therapy and immunotherapy, the inherent immune resistance and high metastasis rates of CM result in significant heterogeneity in treatment outcomes among patients. In BRAF^v600^-mutated CM, commonly used treatment methods for this condition include PD-1 (ProgrammedDeath-1)-based immunotherapy and targeted agents. However, although these treatment methods result in an improvement in survival rates to some extent, typically the 5-year overall survival rate of metastatic CM remains in the range of 40–50%. Additionally, even with the use of these treatment methods, patients who have a high tumor burden, brain metastases, and elevated levels of lactate dehydrogenase (LDH) still generally have a poor prognosis, with a 3-year survival rate below 10%. Consequently, when dealing with CM, there is still a need to investigate more effective treatment methods (Liu et al. 2020). Thus, the identification of effective markers is imperative for enhancing the clinical management of CM and optimizing the rational utilization of medical resources.

Disulfidptosis is a newly discovered form of programmed cell death, first reported by Xiaoguang Liu et al. (2020, 2023). Under conditions of glucose starvation, SLC7A11 (Solute Carrier Family 7 Member 11)-high cells experience reduced intracellular levels of NADPH and accumulated cystine, resulting in aberrant bonding of disulfide that target the actin cytoskeleton and lead to cell collapse. The study also demonstrates that glucose transport protein inhibitors can induce disulfidptosis in tumor cells, inhibiting tumor progression. Overall, this study illustrated the role of disulfidptosis in controlling tumor growth. Cancer cells that have metastasized and exhibit high expression of SLC7A11 are especially vulnerable to oxidative stress. This observation suggests that targeting the cystine apoptosis-related gene could be an effective approach for treating CM.

In this study, we utilized CM data from the TCGA (The Cancer Genome Atlas, https://portal.gdc.cancer.gov/) database to develop and validate a predictive model related to disulfidptosis-related genes (DRGs) for accurately predicting the survival of CM patients. The model demonstrated a significant reduction in the survival rate of the high-risk group in both the training and validation datasets, which exhibits high accuracy in the diagnosis of CM, tumor microenvironment, and the efficacy of immunotherapy.

## Materials and methods

### Data resources

Based on the DRG, this study constructed a signature in CM and downloaded multiple CM cohorts from public databases, as well as immunotherapy cohorts from other cancers. The CM data for this study were obtained from the TCGA database, GTEx (The Genotype-Tissue Expression, https://gtexportal.org/home/) database, GEO (Gene Expression Omnibus, https://www.ncbi.nlm.nih.gov/geo/) database, and TIGER (Tumor Immunotherapy Gene Expression Resource, http://tiger.canceromics.org/#/home) database.

We obtained RNA sequencing (RNA-seq) data, as well as survival data, gender, age, clinical stage, and single nucleotide polymorphism (SNP) data of 473 SKCM patients from the TCGA database. In the GTEx database, we selected RNA-seq data from normal skin tissues. In the GEO database, GSE65904 includes RNA-seq data, survival data, gender, age, and other information of 214 CM patients. GSE54467 includes RNA-seq data, survival data, gender, age, clinical stage, and other information of 79 CM patients. GSE46517 includes RNA-seq data from 104 CM samples and 17 normal samples (including normal skin tissues, nevus tissues, and epithelial melanocytes). In the TIGER database, Melanoma-PRJEB23709 includes RNA-seq data, survival data, immune therapy response data, gender, age, and other information of 91 CM patients. STAD-PRJEB25780 includes RNA-seq data, survival data, immune therapy response data, and other information of 78 gastric cancer patients. In addition, we identified 18 disulfidptosis-related genes from the literature (Liu et al. 2020, 2023) on disulfidptosis.

### Genetic roles and expression analysis of DRGs in CM

For the 18 identified DRGs obtained from the literature(Liu et al. 2020, 2023), we analyzed their roles in CM, including protein–protein interaction (PPI) and enrichment analysis, tumor somatic mutations and copy number variations, as well as differential expression in CM and normal skin tissues.

First, we used the online tool GeneMANIA (https://genemania.org/) for PPI network analysis and data visualization of the 18 DRGs, and performed gene enrichment analysis. Additionally, SNP data were obtained from TCGA (Blum and Wang 2018). The R package “maftools” (Mayakonda et al. 2018) was used to process and analyze the SNP data, counting the occurrences of missense mutations, nonsense mutations, silent mutations, and frameshift/inframe insertions and deletions. Moreover, Tumor Mutational Burden (TMB) was calculated using tumor somatic mutation data. Finally, we selected samples with DRG-associated somatic mutations or copy number variations in CM for analysis and created a waterfall plot.

Lastly, we validated the expression differences of the DRGs in tumor and normal tissues through TCGA-SKCM and GTEx data, as well as the GSE46517 dataset.

### Identifying novel DRGs via unsupervised clustering

We cleaned the missing values in the RNA-seq data and clinical data. Based on the expression of 18 DRGs, we performed cluster analysis on the gene expression data of TCGA-SKCM, GSE65904, and GSE54467 using the R package “ConsensusClusterPlus”. First, we read the expression data file and survival data file. Then, we removed normal samples and preprocessed the data according to the selected grouping criteria, including operations such as transposition, mean subtraction, and logarithmic transformation. These steps ensure the uniformity and comparability of the data. Next, we merged the expression data with the survival data, which helps establish a joint analysis model based on expression data and patient survival time. Then, we conducted univariate COX regression analysis. COX regression models were applied to each gene, and their impact on patient survival was evaluated by calculating p-values, resulting in the selection of significant genes. We then proceeded with cluster analysis. Initially, the maximum number of clusters (maxK) was set to 10, and 50 clustering operations were performed to ensure the stability of the results. We adopted a widely used clustering algorithm (pam) and used Euclidean distance as the clustering metric. The optimal value of K, which provided the highest within-group correlation and lowest between-group correlation, was determined through analysis. Subsequently, the R packages “survival” and “survminer” were used for Kaplan–Meier (KM) analysis of clusters associated with disulfidptosis to compare differences in overall survival (OS). Additionally, we analyzed differences in clinical data and immune cell infiltration levels associated with disulfidptosis clusters. Clinical data included age, gender, staging, T-stage, N-stage, and M-stage, while immune cell infiltration abundance data were obtained through the ssGSEA algorithm, TIMER database, CIBERSORT algorithm, and ESTIMATE algorithm. Differential analysis using the R package “limma” was then performed to identify genes with significant differences between the two groups. The analysis results generated new DRGs.

### Construction and validation of the disulfidptosis-related signature

Based on survival data, a “coxph” function was used to conduct Cox regression analysis on DRGs to identify DRGs significantly associated with survival (P < 0.05). Five machine learning algorithms, including Decision Tree, LASSO, Random Forest, GBDT, and XGBoost, were used to build models and calculate the importance of each DRG in relation to survival. Finally, the results of each model were combined for data fusion to calculate the comprehensive weight of survival correlation.

Subsequently, two R packages, “glmnet” and “survival,” were used to construct the lasso cox model. Firstly, the “cv.glmnet” function was used for ten-fold cross-validation to select the optimal penalty coefficient (λ) for model fitting and further analysis and prediction. Then, the “coef” function was used to extract DRGs associated with non-zero coefficients, and the sum of the product of the coefficients and expression levels of these DRGs represented the risk score. The median risk score of all samples in the training set was used as the cutoff value to divide all samples into high-risk and low-risk groups. KM analysis was performed on the high-risk and low-risk groups, and the R package “timeROC” was used for receiver operating characteristic (ROC) curve analysis to calculate the corresponding area under the curve (AUC) and evaluate the accuracy of the model. Subsequently, external validation of the model was conducted using data from the GEO database and the TIGER cohort.

In addition, to evaluate the relationship between the risk score and clinical data, we analyzed the differences in T stage, N stage, gender, and age between the high-risk and low-risk groups. Furthermore, to better utilize the prognostic model, we constructed a positive determinative graph by integrating the risk score and clinical data.

### Evaluation of the tumor microenvironment in molecular subtypes

The tumor immune microenvironment (TME) plays a crucial role in the occurrence, development, and treatment of cancer. In order to examine the differences in TME between risk subtypes, we obtained evaluations of tumor immune cell infiltration levels from various algorithms based on RNA-seq data from TIMER2.0 (http://timer.cistrome.org/), including TIMER, CIBERSORT, CIBERSORT-ABS, QUANTISEQ, MCPCOUNTER, XCELL, EPIC. We analyzed the differences between the high and low-risk groups. Additionally, the ssGSEA evaluated the infiltration levels of 16 types of immune cells (aDCs, B cells, CD8 + T cells, DCs, iDCs, Macrophages, Mast cells, Neutrophils, NK cells, pDCs, T helper cells, Tfh, Th1 cells, Th2 cells, TIL, Treg) using the R package “GSVA.” We obtained the expression levels of immune checkpoint markers investigated in previous studies and analyzed whether there were differences between the high and low-risk groups. Furthermore, we used the ESTIMATE algorithm to assess the relationship between tumor purity and risk scores, including ESTIMATE Score, Immune Score, and Stromal Score. We also analyzed the immune scores obtained from the TIDE (Tumor Immune Dysfunction and Exclusion, http://tide.dfci.harvard.edu/) online tool.

### Functional enrichment analysis among molecular subtypes

GSEA and GSVA are both methods used to analyze the enrichment of gene sets in gene expression data. In this study, these two methods were used to analyze the pathways and functions associated with the risk model. The KEGG enrichment analysis was conducted using the GSEA software (version 4.2.3). Additionally, we analyzed the correlation between risk scoring and pathways related to disulfidptosis as well as tumor-related pathways.

The disulfidptosis-related pathways include: functions related to cytoskeletal organization, pentose phosphate pathway, lipid homeostasis, glutathione metabolism, tricarboxylic acid cycle, P53 signaling pathway, glutathione peroxidase activity, Nrf2 signaling pathway, cell response to glucose starvation, calcium binding involved in cell adhesion, cell adhesion molecule binding, and calcium binding. Important signaling pathways in tumors include Hippo, Wnt, MAPK, PI3K/AKT, TGF-β, NF-kB, Notch, AMPK, JAK-STAT, PD-1/PD-L1, mTOR, Ras, TNF, HIF-1, and ErbB.

### Antibodies and reagents

Five monoclonal antibodies anti-HLA-DQA1 antibody (1:10,000 dilution for WB, 1:200 dilution for IHC, ab128959), anti-GZMA antibody (1:1000 dilution, ab209205), anti-CD79A antibody (1:10,000 dilution, ab79414), anti-LTB antibody (1:1000 dilution, ab89568) and anti-HE5 antibody (1:1000 dilution, ab125071) were purchased from Abcam (Cambridge, UK). Monoclonal antibody anti-β-actin (1:20,000 dilution, 66009-1-Ig) was purchased from Proteintech (Wuhan, China).

### Cell culture and transfection

The human immortalized keratinocyte HaCaT, the human malignant melanoma cell line A-375, A-875 and SK-MEL-1 was obtained from Haixing Biosciences, Suzhou, Jiangsu, China. Cells were cultured in Dulbecco's Modified Eagle’s Medium (Gibco, Invitrogen, Paisley, UK) supplemented with10% fetal bovine serum and 1% streptomycin-penicillin (Gibco, Invitrogen, Paisley, UK) at 37 °C in a humified 5% CO2 incubator. Lipofactamine3000 (Thermo Fisher Scientific, Waltham, MA, USA) was used to transfect cells with siRNAs (RiboBio, Guangzhou, China following the manufacturer’s guidelines. 5′-GATGGAGATGAGCAGTTCT′ (st-h-HLA-DQA1_001), 5′-CTTGTGGTGTAAACTTGTA-3′ st-h-HLA-DQA1_002), and 5′-CAACTTGAACATCATGATT-3′ (st-h-HLA-DQA1_003) were the three target sequences for siRNA for HLA-DQA1.

### Real-time quantitative PCR assay

Total RNA was extracted from HaCaT and the three the human malignant melanoma cell lines, using NucleoZOL reagent (740404.200, Meilunbio). Total RNA was then reverse transcribed into cDNA using Evo M-MLV RT Kit with gDNA Clean for qPCR (AG11705, AG, Hunan, China). Real-time quantitative PCR (RT-qPCR) was performed using the SYBR Green Premix Pro Taq HS qPCR Kit IV (AG11746, AG, Hunan, China). Relative quantification was determined using the 2(−ΔΔCt) method.

### Western blotting analysis

The cell precipitate was lysed with RIPA (C5029, Bioss) to extract the total protein. The protein concentration was measured using the BCA method. Around 25 μg of total protein underwent gel electrophoresis and was transferred to a PVDF membrane (Millipore, Kenilworth, NJ, USA). Afterwards, the membrane was blocked with Protein-Free Rapid Blocking Buffer (Yamei, Shanghai, China) at room temperature for 15 min. The membrane was then incubated overnight at 4 °C with the primary antibody. The next day, the membrane was washed three times with TBST for 5 min each, and then incubated with the secondary antibody and membrane at room temperature for 1 h. Detection was carried out using the Immobilon Western Chemilum HRP Substrate Kit (WBKL S0500, Millipore, Kenilworth, NJ, USA). Specific areas of the gel were scanned to acquire images, and Adobe Photoshop software (CS4, Adobe Systems, USA) and ImageLab software (Bio-Rad, USA) were used for quantification.

### Immunohistochemistry analysis

The tissue microarray chip (HMelC112CD01, Gongxie, Shandong, China) in the CM group underwent immunohistochemistry (IHC) detection of anti-HLA-DQA1 antibody following the standard labeled streptavidin biotin (LSAB) protocol (Dako, Carpinteria, CA). The immune reactive score (IRS) for each sample was calculated by multiplying the positive cell percentage score (0: no positive cells, 1: < 10%, 2: 10–50%, 3: 51–80%, 4: > 80%) with the staining intensity score (0: no color reaction, 1: mild reaction, 2: moderate reaction, 3: strong reaction). Immunohistochemical staining of tissue chips was performed using a two-step method. When the HLA-DQA1 score in cancer tissue was higher than that in non-tumor tissue, the expression level of GS in cancer tissue was considered upregulated (high); all other scores were considered down-regulated (low).

### Cell proliferation assay

Cell proliferation was analyzed using the Cell Counting Kit-8 (BIOSS, Beijing, China) according to the manufacturer’s protocol. The melanoma cells transfected with siRNA expressing HLA-DQA1 and empty vector were diluted and seeded at a density of 1 × 10^3^ cells per well in a 96-well plate containing 0.1 mL of culture medium. After 24 h of incubation, each well was treated with 10 μl of CCK-8 solution at 37 °C. Subsequently, the absorbance at 450 nm was measured using the Multiskan SkyHigh (Thermo Fisher, Stony Creek, the US).

### Clonogenic assay

Gather the a-375 and A-875 cell lines with downregulated HLA-DQA1 and the control group cells, and seed 600 cells per well in a 6-well plate. The plate was then incubated at 37 ℃ for a duration of 14 days. Each well was treated with 500 μl of paraformaldehyde and fixed at room temperature for 25 min. D The paraformaldehyde was then discarded and the wells were washed with PBS. Next, 400 μl of a 2.5% methanol-crystal violet staining solution was added to each well and stain for 15 min. Finally, the observed cells were inspected and the number of cell clones was counted under a microscope.

### Cell migration and invasion assay

Corning’s 80 μm 24-well Transwell plates (Falcon) were coated with 30% Matrigel (300 μl/well) for use in migration and invasion assays. Each upper chamber of the Transwell plates received 1 × 10^5^ cells in 100 μl of serum-free medium, while the lower chamber was filled with 600 μl of medium containing 20% fetal bovine serum. After a 6-h incubation at 37 °C, non-migrating cells that remained in the upper chamber were gently removed from the upper surface of the Matrigel using a cotton swab. Subsequently, the cells were stained with 2.5% methanol-crystal violet solution for 15 min and subsequently enumerated. Five random microscopic fields (× 100 magnification) were assessed per well, and the mean was calculated.

### Statistical analysis

In this study, a variety of statistical and bioinformatics analysis methodologies were employed. We initially conducted Wilcoxon rank-sum tests to compare the differences between two groups of samples. This choice was based on the test’s suitability for analyzing non-normally distributed data. Furthermore, Pearson’s Chi-squared test was used to assess the association between categorical variables in the dataset.

Additionally, t-tests were employed to examine the significance of differences between means in normally distributed data. For analyzing differences among sample groups containing more than two samples, the Kruskal–Wallis test was utilized as a non-parametric alternative to one-way ANOVA.

The threshold for statistical significance throughout the analysis was set at P < 0.05, indicating that P-values less than 0.05 were considered statistically significant. All statistical analyses were conducted exclusively using the R programming language. R is well-suited for bioinformatics data analysis due to its extensive ecosystem of packages and functions.

## Results

### Genetic characteristics and transcriptional changes of DRGs in CM

**Fig. 1:**
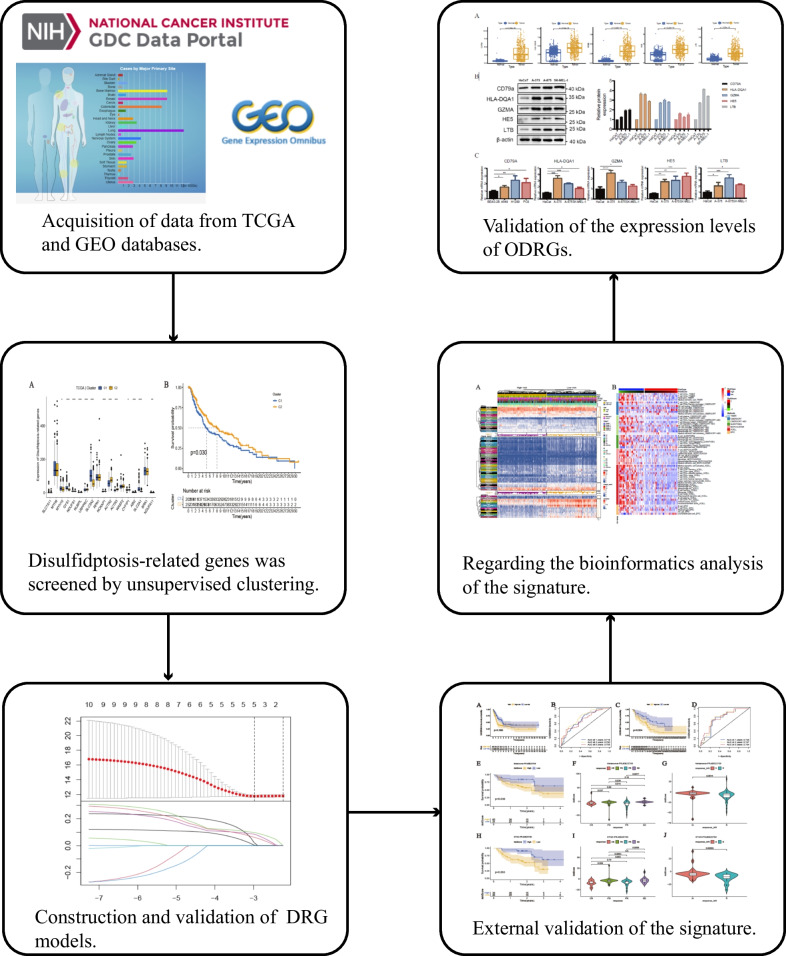
The workflow of this study. The figure illustrates the process of constructing and subsequent analysis of the DRG model

**Fig. 2:**
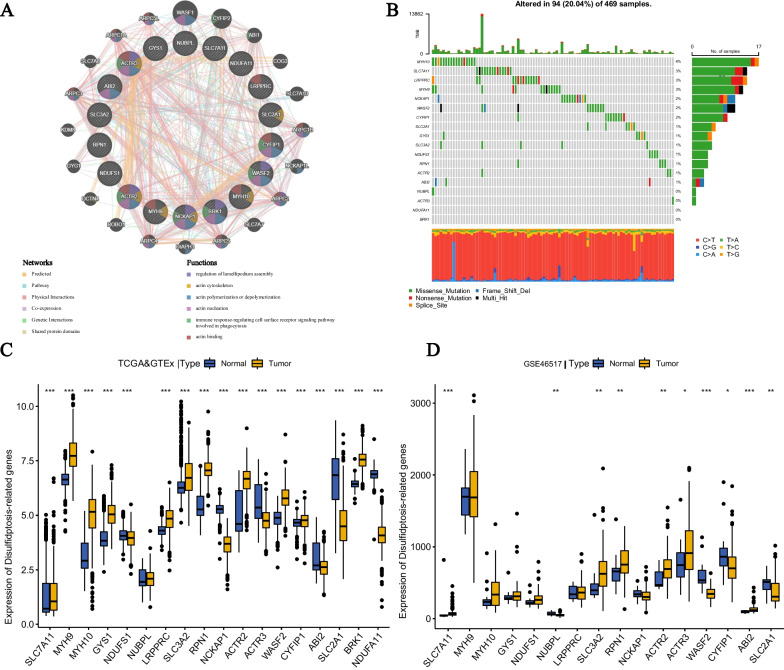
Genetic characteristics and transcriptional changes of disulfidptosis related genes in CM. **A** Network diagram of PPI analysis and enrichment analysis of 18 DRGs. **B** Somatic mutation and copy number variation (CNV) information for all 18 DRGs within the TCGA-SKCM dataset. **C** The differential expression of 18 DRGs between CM patients and normal skin tissues was analyzed in the TCGA and GTEx datasets. **D** The differential expression of 18 DRGs between CM patients and normal skin tissues was analyzed in the GSE46517 dataset. *P < 0.001

The specific procedure of this study is depicted in Fig. 1. First, we constructed a protein–protein interaction (PPI) analysis network of 18 DRGs using the online tool GeneMANIA to explore their interactions. The enriched functions mainly include regulation of lamellipodium assembly, actin cytoskeleton, actin polymerization or depolymerization, actin nucleation, immune response-regulating cell surface receptor signaling pathway, involved in phagocytosis, and actin binding (Fig. 2A). Next, we analyzed somatic mutations and CNV (copy number variation) of CM using TCGA-SKCM SNP data. The somatic mutation patterns of the 18 DRGs were evaluated (Fig. 2B). The results showed that 94 out of 469 CM patients (20.04%) were regulated by DRG mutations. The predominant type of mutation was missense mutation, mainly occurring in MYH10, SLC7A11, LRPPRC, MYH9, NCKAP1, WASF2, and CYFIP1. Somatic mutations were predominantly concentrated in MYH10. Furthermore, we analyzed the expression levels of the 18 DRGs in CM samples and normal samples from the TCGA&GTEx and GSE46517 datasets (Fig. 2C, D). Key genes in disulfidptosis, such as SLC7A11, were highly expressed in CM tissues. Additionally, MYH9, MYH10, GYS1, LRPPRC, SLC3A2, RPN1, and ACTR2 were highly expressed in CM, while NDUFS1, NUBPL, NCKAP1, and SLC2A1 were downregulated in CM.

### Identification of novel disulfidptosis-related genes

**Fig. 3:**
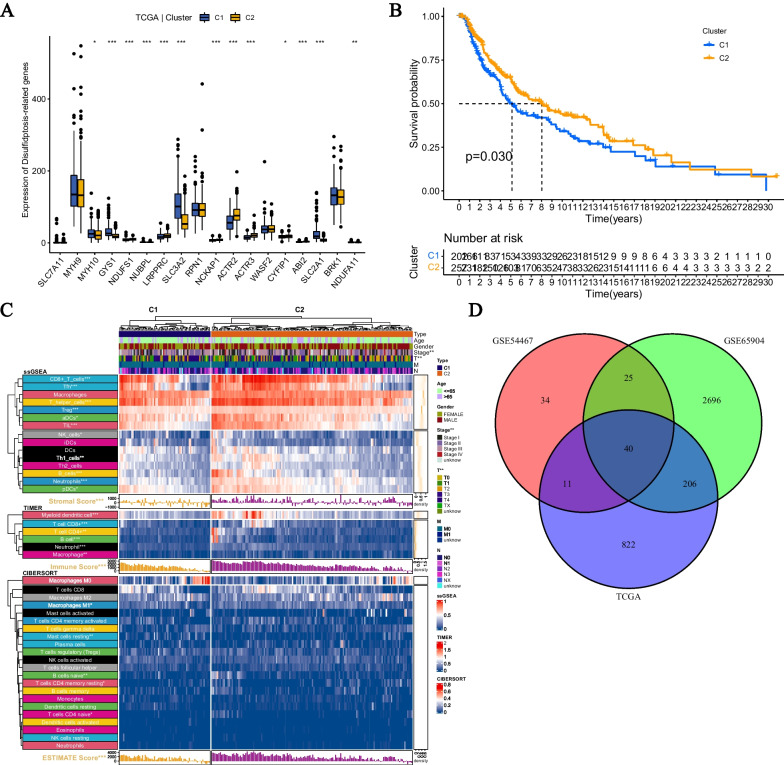
Screening of DRGs through disulfidptosis clustering. **A** Differential gene expression of disulfidptosis-related genes between two disulfidptosis-related clusters. **B** Kaplan–Meier survival curves of patients in the two clusters. **C** Differential abundance of immune cell infiltration in DRG-related clusters. **D** By performing unsupervised clustering on the TCGA dataset, GSE65904, and GSE54467, a total of 40 DRGs were obtained

In order to explore the expression characteristics of DRGs in CM, we clustered TCGA-SKCM based on the RNA-seq data of 18 DRGs using consensus clustering. The results showed that the stability was optimal when K = 2. We further investigated the clusters related to disulfidptosis and analyzed the expression levels and overall survival of the 18 DRGs. In TCGA-SKCM, there were differences in the expression levels of most DRGs between clusters (Fig. 3A). The expression of MYH10, GYS1, SLC3A2, SLC2A1, and NDUFA11 was higher in cluster 1 compared to cluster 2, while NDUFS1, NUBPL, LRPPRC, NCKAP1, ACTR2, ACTR3, CYFIP1, and ABI2 showed the opposite trend. In terms of survival, the overall survival of cluster 1 was lower than that of cluster 2, and there was a statistical difference (Fig. 3B).

In addition, we further studied the clusters related to disulfidptosis and analyzed the clinical characteristics, immune scores, and immune cell infiltration abundance between the two disulfidptosis-related clusters. In terms of clinical characteristics, there were differences in clinical stage and T stage between the two disulfidptosis-related clusters. As for immune cell infiltration data, there were differences in CD8 + T cells, Tfh cells, T helper cells, Treg cells, aDCs, TIL, NK cells, B cells, Neutrophils, and pDCs in the ssGSEA algorithm; Myeloid dendritic cells, T cell CD8 +, T cell CD4 +, B cells, Neutrophils, and Macrophages in the TIMER algorithm; Mast cells resting, B cells naive, T cells CD4 memory resting, and T cells CD4 naive in the CIBERSORT algorithm. Additionally, there were significant differences in ESTIMATE Score, Immune Score, and Stromal Score between the two disulfidptosis-related clusters through ESTIMATE analysis.

Through the above analysis, we have demonstrated the significance of our clustering of TCGA-SKCM. In order to screen for new DRGs, we conducted differential analysis of the clusters related to disulfidptosis according to the criteria (|log2FC|≥ 1, P < 0.05). To further narrow down our selection, we performed consensus clustering of GSE65904 and GSE54467, and analyzed the differentially expressed genes. By taking the intersection of the differentially expressed genes in the three datasets, we obtained a total of 40 new DRGs.

### The development of disulfidptosis-related signature in CM

**Fig. 4:**
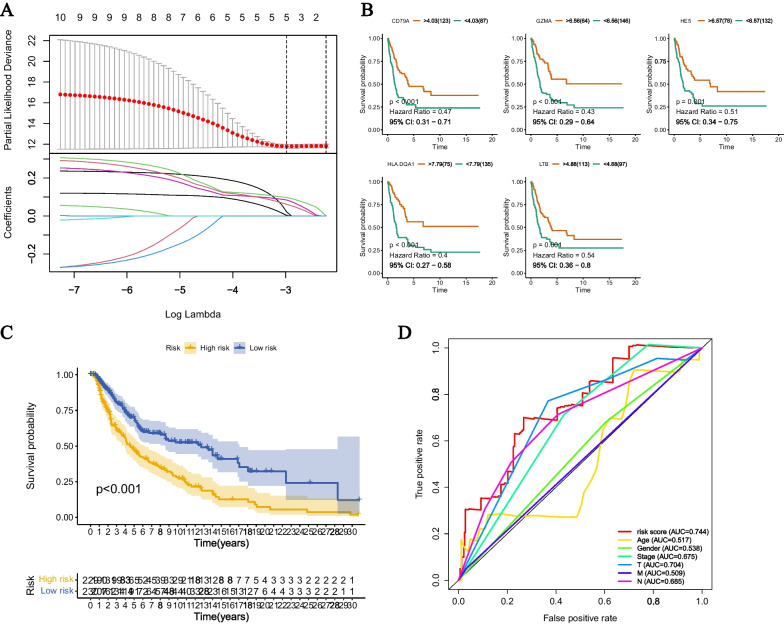
Screening and model construction of ODRG. **A** The best λ value was selected through LASSO regression and LASSO coefficients were configured. **B** The survival rates of the high-expression group and low-expression group (i.e. CD79B, GZMA, HE5, HLA-DQA1, and LTB) of ODRGs showed significant differences. **C** Survival differences between high-risk and low-risk groups in the TCGA cohort. The table below shows the number of patients still alive each year. **D** The ROC curve demonstrates the predictive efficiency of risk score and clinical features

In order to construct a prognostic model, we used univariate Cox analysis to select 38 DRGs associated with survival. The weights of the prognostic-related DRGs in terms of survival were evaluated using algorithms such as Decision Tree, LASSO, Random Forest, GBDT, and XGBoost. The top ten genes (Table 1) were selected to build the LASSO Cox model (Fig. 4A). The genes used in constructing the model displayed KM curves for high and low expression groups (Fig. 4B). The risk score is calculated as follows: (− 0.0787 × exp (CD79A)) + (0.3804 × exp (HE5)) + (− 0.15832 × exp (HLA-DQA1)) + (− 0.2650 × exp (GZMA)) + (− 0.0888 × exp (LTB)). Subsequently, based on the median of the risk score, patients with CM were divided into high and low-risk groups. The high-risk group has a higher probability of death compared to the low-risk group (Additional file 1: Figure S1A). PCA and t-SNE plots demonstrate the distinct distribution of high and low-risk groups (Additional file 1: Figure S1B). The Kaplan–Meier survival curve shows a significantly worse overall survival (OS) for high-risk CM patients compared to low-risk group (Fig. 4C, P < 0.001). The predictive performance of this prognosis model was evaluated by ROC curve analysis, with an AUC value of 0.744. Furthermore, through univariate and multivariate Cox analysis, we demonstrated that age (HR = 1.011, 95% confidence interval (CI) = 1.000–1.022, P = 0.047), T stage (HR = 1.457, 95% CI = 1.232–1.722, P < 0.001), N stage (HR = 1.590, 95% CI = 1.261–2.004, P < 0.001), and risk score (HR = 2.389, 95% CI = 1.679–3.400, P < 0.001) are independent prognostic factors for CM (Table 2).

**Table 1:** The quantified importance of prognostic disulfidptosis-related messenger genes by machin learning

	Decision Tree	LASSO	Random Forest	GBDT	XGBoost	AVG	
GBP5	0.013827	0.373494	0.215771	0.165928	0.044820	0.162768	(1)
HLA-DQA1	0	0.229167	0.149802	0.112942	0.029389	0.10426	(2)
HLA-DRA	0.069321	0	0.217371	0.202672	0.021325	0.102138	(3)
CD79A	0.043209	0.08349	0.15570	0.074456	0.022523	0.075875	(4)
HE5	0.054514	0.280299	0.023088	0.015352	0.025615	0.070538	(5)
HLA-DMB	0.060051	0.023476	0.115609	0.083555	0.012552	0.059049	(6)
LTB	0.064557	0	0.026341	0.025064	0.027094	0.0286111	(7)
CD2	0.418228	0	0.035964	0.032222	0.025557	0.027113	(8)
GZMA	0.034531	0	0.041947	0.025454	0.030842	0.026555	(9)
CCL5	0.024580	0	0.02469	0.059022	0.012451	0.024148	(10)

The number in the parentheses represented the rankings of weight

**Table 2:** Independent analysis of training set patients

Characteristics	Univariate			Multivariate		
	HR	95%CI	P	HR	95%CI	P
Age	1.019	1.009–1.030	< 0.001	1.011	1.000–1.022	0.047
T stage	1.479	1.278–1.711	< 0.001	1.457	1.232–1.722	< 0.001
N stage	1.440	1.239–1.674	< 0.001	1.590	1.261–2.004	< 0.001
Risk score	2.807	1.958–4.023	< 0.001	2.389	1.679–3.400	< 0.001

HR: hazard ratio; CI: confidence interval

### Validation of signature and prediction of immunotherapy response

**Fig. 5:**
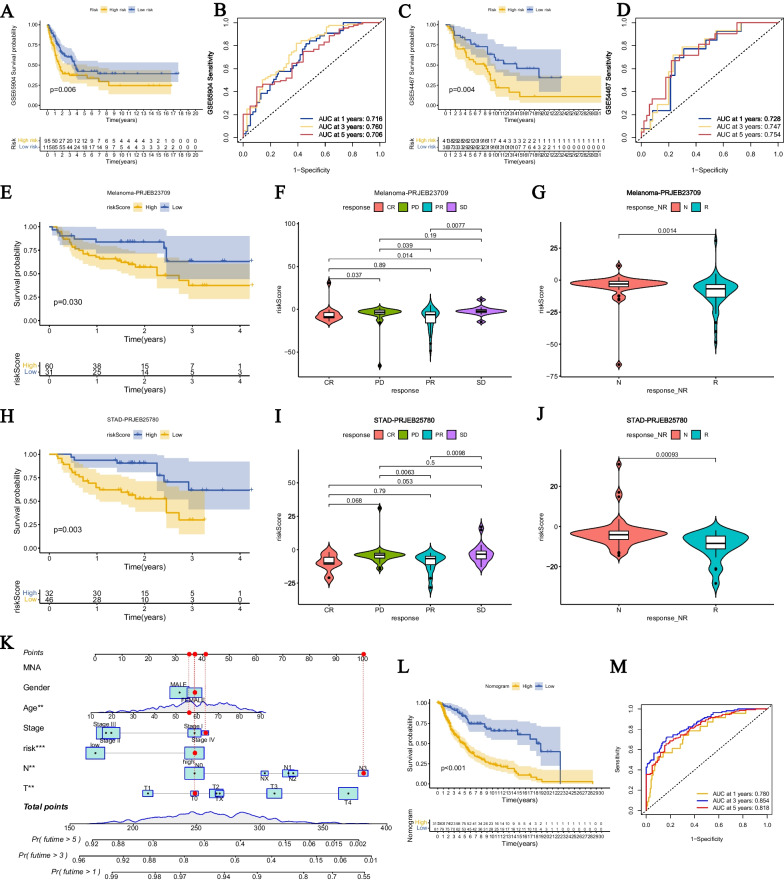
Validation of the risk model. **A**, **C** Survival curves of the high-risk group and low-risk group in the validation set (GSE65904, GSE54467). **B**, **D** AUC values of the ROC curves for risk scores in the validation set (GSE65904, GSE54467). Difference in the immunotherapy efficacy between the high-risk and low-risk groups in **E** Melanoma-PR-JEB23709 and **H** STAD-PRJEB25780. Comparison of risk scores after immunotherapy in different CM patients with different remission statuses in **F** Melanoma-PRJEB23709 and **I** STAD-PRJEB25780. Comparison of risk scores after immunotherapy in different reaction statuses of CM patients in **G** Melanoma-PRJEB23709 and **J** STAD-PRJEB25780. **K** Nomogram depicting risk scores and clinical indicators. The red line represents an example of how prognosis is predicted. **L** Survival differences between the high and low score groups in the column line plots. **M** ROC curves demonstrate the predictive efficiency of the nomogram.*P < 0.05; **P < 0.01; ***P < 0.001

To validate the repeatability of disulfideptosis-associated signatures in CM and their application in immunotherapy, we conducted external validation using the GSE65904, GSE54467 datasets from the GEO database, as well as the Melanoma-PRJEB23709 and STAD-PRJEB25780 datasets from the TIGER database. The results of KM survival analysis showed that in GSE65904 and GSE54467, patients with high-risk CM had significantly lower overall survival (OS) than patients with low-risk CM (Fig. 5A, C). The ROC curves performed well, with AUC values of 0.716, 0.760, and 0.706 for 1, 3, and 5 years in GSE65904, and AUC values of 0.728, 0.747, and 0.754 for 1, 3, and 5 years in GSE54467 (Fig. 5B, D). In the immunotherapy data from TIGER, patients with high-risk CM in Melanoma-PRJEB23709 had lower OS than patients with low-risk CM (Fig. 5E). We also compared the risk scores of patients in different states during CM treatment. Patients with complete response (CR) had lower risk scores than patients with disease progression (PD) and disease stability (SD), while patients with partial response (PR) had lower risk scores than PD and SD patients (Fig. 5F). Moreover, the risk scores of patients who responded to immunotherapy were significantly lower than those of patients who did not respond (Fig. 5G). In STAD-PRJEB25780, patients in the high-risk group had significantly lower OS than those in the low-risk group (Fig. 5H). The risk scores of CR and PR patients were lower than those of PD and SD patients (Fig. 5I), and for patients who responded to immunotherapy, their risk scores were significantly lower, while for those who did not respond to immunotherapy, the risk scores were higher (Fig. 5J). Furthermore, we constructed a nomogram combining the risk score and clinical features (Fig. 5K). Patients with lower scores in the nomogram had significantly higher overall survival (OS) rates than those with higher scores (Fig. 5L). Moreover, the AUC values for 1-year, 3-year, and 5-year in the time-dependent ROC curve were 0.78, 0.854, and 0.818(Fig. 5M), respectively, indicating a good predictive performance.

### Analysis of the clinical features of risk subtypes

**Fig. 6:**
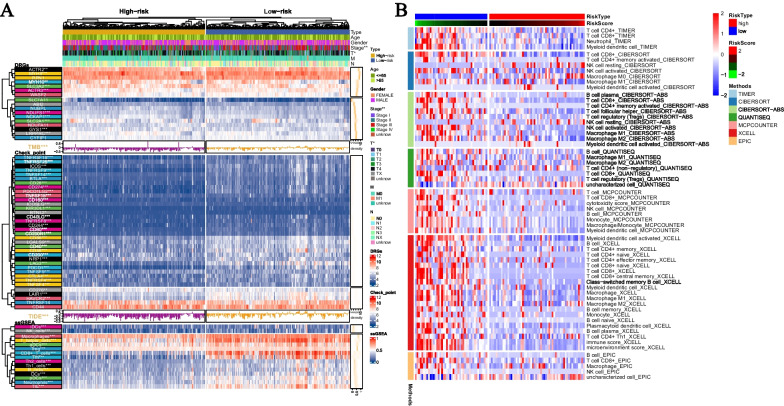
Immune infiltration analysis of ODRGs. **A** Differences in clinical data, DRG, TMB, TIDE scores, and ssGSEA results between high-risk and low-risk groups. TIDE scores and TMB are presented in the form of bar graphs and density plots, respectively. **B** Abundance differences of immune cell infiltration algorithms (such as TIMER, CIBERSORT, CIBERSORT-ABS, MCPCOUNTER, XCELL, and EPIC) between high-risk and low-risk groups are presented in a heatmap (displaying statistical differences)

We analyzed the differences between risk scores and clinical data, and the results showed that there were differences in clinical stage and T stage between the two groups (Fig. 6A). In addition, the risk scores obtained from the disulfidoptosis-related prognostic model were also associated with the expression of DRG. There were differences in the expression of ACTR2, MYH9, SLC3A2, ACTR3, NDUFS1, NCKAP1, SLC2A1, GYS1, and LRPPRC between the high-risk and low-risk groups (Fig. 6A).

### Analysis of immune cell infiltration abundance and immune scores

This study analyzed the association between immune scores, immune checkpoints, immune cell infiltration abundance, and the disulfidptosis-related prognostic model. The results showed differences in TMB and TIDE between the high and low-risk groups (Fig. 6A). The expression levels of 26 immune checkpoints, including CTLA4, CD274 (PD-L1), PDCD1 (PD1), PDCD1LG2 (PD-L2), varied between the high and low-risk groups (Fig. 6A). In the ssGSEA algorithm, differences in infiltration abundances of iDCs, NK cells, Macrophages, T helper cells, aDCs, Treg, CD8 + T cells, Tfh, Th2 cells, Th1 cells, B cells, DCs, pDCs, Neutrophils, and TIL were observed between the high and low-risk groups (Fig. 6A). Additionally, the levels of immune cell infiltration that showed statistical differences between the high and low-risk groups were demonstrated in the TIMER database data (Fig. 6B).

### Pathway and functional analysis

**Fig. 7:**
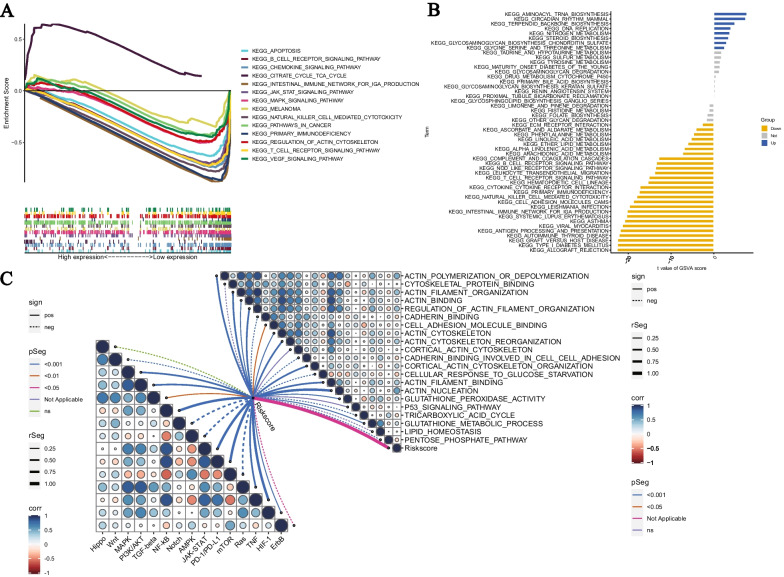
Biological Functions. **A** Pathways significantly enriched in high-risk and low-risk populations. Extreme values on the left indicate a positive correlation between risk scores and pathway activity, and vice versa. **B** Significant differences exist in the pathways between the high-risk and low-risk groups. Blue bars represent a positive correlation between risk scores and pathway activity, while yellow bars represent the opposite. **C** Correlation between risk scores and tumor-specific pathways and functions related to disulfidptosis

Biological analysis using GSEA software showed that the citrate cycle tca cycle is active in the high-risk group, while apoptosis, B cell receptor signaling pathway, chemokine signaling pathway, citrate cycle tca cycle, intestinal immune network for IGA production, JAK STATsignaling pathway, MAPK signaling pathway, melanoma, natural killer cell mediated cytotoxicity, pathways in cancer, primary immunodeficiency, regulation of actin cytoskeleton, T cell receptor signaling pathway, VEGF signaling pathway are active in the low-risk group (Fig. 7A). The results of GSVA enrichment analysis showed that DNA replication, aminoacyl tRNA biosynthesis, and other functions are enriched in the high-risk population, while antigen processing and presentation, intestinal immune network for IGA production, cell adhesion molecules cams, natural killer cell mediated cytotoxicity, primary immunodeficiency, cytokine cytokine receptor interaction, T cell receptor signaling pathway, leukocyte transendothelial migration, nod like receptor signaling pathway, B cell receptor signaling pathway, complement and coagulation cascades, arachidonic acid metabolism, ether lipid metabolism, linoleic acid metabolism, phenylalanine metabolism, ECM receptor interaction are active in the low-risk group (Fig. 7B). In addition, important tumor pathways such as Wnt, MAPK, PI3K/AKT, TGF-beta, NF-kB, Notch, AMPK, JAK-STAT, PD-1/PD-L1, mTOR, Ras, TNF, HIF-1, ErbB are also associated with the risk score, and pathways and functions are correlated as well. The factors associated with disulfidptosis are also correlated with risk scoring (Fig. 7C).

### Validates the role of the key gene HLA-DQA1 in melanoma cell lines in vitro

**Fig. 8:**
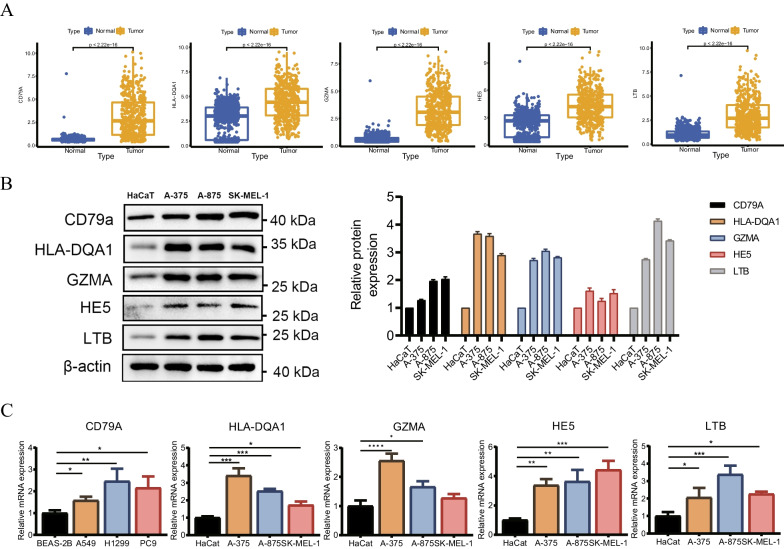
Verification of ODRGs expression. **A** Distribution of the DRG score between normal and tumor tissue. **B** Western Blotting verifies the expression of ODRGs in 1 normal cell strain and three types of melanoma cells. **C** PCR verification OCIRGS’s expression level

**Fig. 9:**
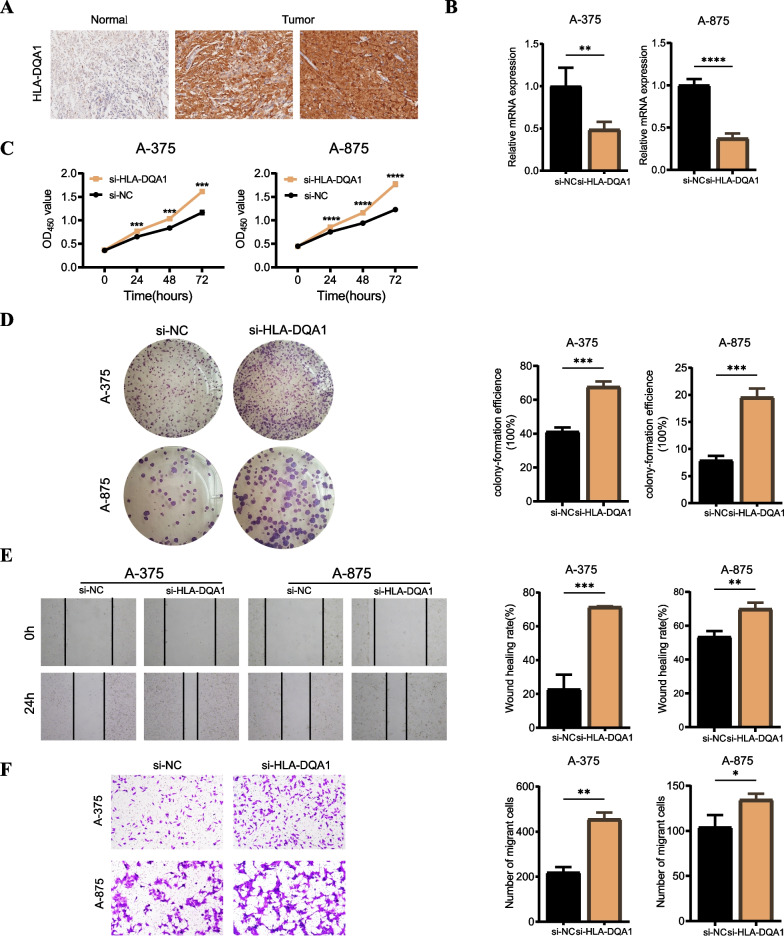
Validates the role of the key gene HLA-DQA1 in melanoma cell lines in vitro. **A** Representative IHC images of HLA-DQA1 protein expression in CM and benign nevus tissues. **B** Knockdown of HLA-DQA significantly reduced its expression in A-375 and A-875 cell lines (**P < 0.01, ****P < 0.0001). **C** After HLA-DQA1 knockdown in A-375 and A-875 cell lines, the increase of cell proliferation was markedly enhanced (***P < 0.01, ****P < 0.0001). **D** Clonogenic assays showed a significant increase in the ability of A-375 and A-875 cell lines to form colonies after HLA-DQA1 knockdown (***P < 0.001). **E** The si-NC group in the wound healing experiment of A-375 and A-875 cell lines showed weaker migration ability than the si-HLA-DQA1 group (**P < 0.01, ***P < 0.001). **F** The invasion ability of A-375 and A-875 cell lines significantly increased after HLA-DQA1 knockdown (*P < 0.5, **P < 0.01)

Using patient data from the TCGA database, we initially validated the expression levels of 5 ODRGs in both normal tissues and melanoma (Fig. 8A). We then validated the expression levels of these genes in HaCaT cells and three melanoma cell lines through q-PCR and Western Blot analysis (Fig. 8B, C). HLA-DQA1 showed the highest regression coefficient among the genes analyzed in our risk model. Consequently, we chose HLA-DQA1 as a candidate gene and examined its functional role in CM. A-375 and A-875 cells were transfected with siRNA that targeted HLA-DQA1. Figure 9A shows a significant reduction in the expression of HLA-DQA1 in A-375 and A-875 cell lines compared to HaCaT cells. Subsequent experiments were conducted to evaluate the effects of inhibiting HLA-DQA1 expression on melanoma cell proliferation, invasion, and migration. The results showed that knocking down HLA-DQA1 substantially improved the survival of melanoma cells compared to the control group, as illustrated in Fig. 9B and C. Moreover, the downregulation of HLA-DQA1 significantly enhanced melanoma cell invasion, as illustrated in Fig. 9D. In addition, the scratch closure rate of melanoma cells transfected with siHLA-DQA1 was significantly higher than that of the control group after 24 h of plating, suggesting a significant enhancement in their migratory ability (Fig. 9E). These findings indicate differential expression of HLA-DQA1 in both HaCaT and melanoma cell lines, and its regulation of melanoma cell proliferation, invasion, and migration.

## Discussion

CM is a highly invasive and deadly skin cancer. In recent years, emerging technologies such as targeted therapy and immune checkpoint inhibitors have brought new hope to the treatment of CM. Clinical trials play a crucial role in evaluating efficacy and safety. However, we still face many challenges. Firstly, our understanding of the etiology and pathogenesis of CM is insufficient, necessitating further research to uncover its underlying causes in order to better formulate treatment strategies. Additionally, due to the complexity and heterogeneity of CM, it is crucial to identify new biomarkers to guide clinical treatment (Guo et al. 2021; Berk-Krauss et al. 2020; Luke et al. 2017). Our goal is to conduct in-depth research to seek new treatment methods and biomarkers for more accurate diagnosis and prediction of treatment response in CM, thereby achieving more personalized treatment. Despite some progress, further research is still needed for a comprehensive understanding of CM and to provide more effective guidance for clinical treatment. Recent studies have recently discovered a novel form of cell death, known as disulfidptosis (Liu et al. 2023), which may hold potential therapeutic value and possibilities for targeted cancer treatment. Consequently, biosignatures based on genes associated with disulfidptosis could potentially open up a new research field.

In this study, unsupervised clustering was conducted on TCGA-SKCM to identify clusters related to disulfidptosis. The reliability of this grouping was confirmed from multiple perspectives, including the expression of DRG, overall survival (OS), clinical characteristics, and levels of immune cell infiltration. In order to select more accurate DRGs, we also incorporated GSE54467 and GSE65904. Subsequently, we used univariate COX and multivariate COX analysis to screen for prognosis-related genes and used lasso to identify 5 ODRGs for constructing proportional hazard regression models. This signature accurately predicted the survival and immunotherapy response of patients with cutaneous CM. Compared to the high-risk group, the low-risk group had higher survival and immunotherapy sensitivity. In addition, the nomogram constructed by combining the risk score and clinical characteristics showed good accuracy and stability.

In 5 ODRGs, CD79A (CD79a molecule) is a protein closely related to the development and function of B cells. Studies have found that CD79A plays an important role in oral squamous cell carcinoma (OSCC) and may serve as a potential biomarker for treatment and prognosis (Yao et al. 2023). Through gene expression analysis and clinical data, researchers have found that high expression of CD79A is associated with better prognosis in oral squamous cell carcinoma patients. Further studies have shown that CD79A is enriched in the B cell receptor signaling pathway, and high levels of infiltration of immature B cells are associated with better prognosis, while high levels of infiltration of memory B cells are associated with poorer prognosis. These findings are of significant importance for the treatment and prognosis evaluation of oral squamous cell carcinoma.

GZMA (Granzyme A) is a protein produced by cytotoxic lymphocytes that plays an important role in immune reactions. Recent studies have shown that GZMA plays a significant role in hepatocellular carcinoma (HCC) and breast cancer. In HCC patients, the expression level of GZMA is closely associated with tumor growth, metastasis, and prognosis. Patients with low GZMA expression usually have larger tumor burden and malignant cell characteristics, resulting in poorer prognosis (Gao et al. 2022). Further research has revealed that GZMA interacts with the F2R receptor on the surface of tumor cells, activating the JAK2/STAT1 signaling pathway, inducing tumor cell apoptosis, and T cell-mediated tumor killing. Similarly, the expression of GZMA in breast cancer is associated with malignant features, while low GZMA expression is related to higher tumor grade, lymph node metastasis, and worse prognosis (Huo et al. 2023). In summary, GZMA plays an important role in hepatocellular carcinoma and breast cancer. Understanding its mechanisms of action can contribute to the development of novel immunotherapy strategies and improve patient prognosis.

According to the latest research, HE5 plays an important role in the treatment of mantle cell lymphoma and breast cancer. Studies on mantle cell lymphoma have shown that the expression of HE5 increases in MCL cells when treated with ibrutinib (Fuhr et al. 2022). Co-administration of HE5 monoclonal antibody (mAb) and ibrutinib enhances the toxic effect on ibrutinib-sensitive MCL cells. Treatment with HE5 mAb and human serum after pretreatment leads to a reduction and dissolution of MCL cells. In breast cancer research, HE5 has been found to be significantly increased in breast cancer patients and is associated with a better prognosis (Ma et al. 2021). Particularly in early-stage breast cancer, HE5 expression is higher and correlates with immune cell infiltration, T cells, and M1 macrophages. HE5 may have an inhibitory effect on breast cancer and participate in the regulation of immune cell types and numbers.

HLA-DQA1 (MHC II HLA-DQ-Alpha-1) is a type of HLA-II gene located on human chromosome 6, which plays an important role in the immune system by presenting antigen fragments of extracellular proteins to activate T cells and participate in immune responses. Studies have shown that HLA-DQA1 is closely associated with the development and prognosis of multiple tumors, having significant clinical value. For example, in lung adenocarcinoma, the DQA1*03 allele is closely related to the risk of developing lung adenocarcinoma, providing new clues for risk assessment and personalized treatment of lung adenocarcinoma (Kohno et al. 2010). In the case of esophageal cancer, high levels of HLA-DQA1 expression are associated with clinical characteristics and prognosis of esophageal squamous cell carcinoma (Shen et al. 2019). In oral cancer and breast cancer, HLA-DQA1 polymorphism is related to incidence, and high levels of expression are associated with better prognosis, offering potential biomarkers for prediction and treatment of oral cancer and breast cancer (Tsai et al. 2011; Zhou et al. 2023; Mahmoodi et al. 2012). Furthermore, imaging models have shown promising predictive performance in predicting HLA-DQA1 expression levels, providing a basis for personalized prediction of HLA gene expression. Considering the limitations and potential off-target effects of siRNA, during the experimental validation process, researchers screen for specific guide RNA (gRNA) during the initial selection process to address the limitations of siRNA and reduce potential off-target effects. Additionally, eSpCas9 was utilized to increase delivery efficiency and minimize off-target effects. In summary, HLA-DQA1 plays an important role in various tumors, with clinical value in assessing the risk, predicting prognosis, and personalized treatment of tumors. However, further research is still needed to validate these findings and elucidate the underlying mechanisms of HLA-DQA1 in tumor development.

LTB (Lymphotoxin β) is a cell surface molecule that plays an important role in the immune system. Recent research has found that LTB has potential value in the treatment of various tumors. Studies have shown that in CM, activating LTB receptors can inhibit melanoma cell proliferation and induce the release of inflammatory chemokines, potentially promoting the treatment of CM and triggering immune responses (Degli-Esposti et al. 1997). In the case of liver cancer, activating LTB receptors can inhibit tumor cell proliferation and migration, induce cell apoptosis, and promote the activation of tumor anti-tumor immune responses, thereby improving the treatment efficacy of liver cancer (Subrata et al. 2005). In head and neck tumors, research has found that LTB can inhibit cell proliferation and invasion, and induce cell apoptosis (Das et al. 2019). Furthermore, LTB can also suppress the growth and metastasis of head and neck tumors by activating the immune system. Finally, studies have found a close correlation between LTB and the loss of follicular dendritic cell (FDC) phenotype in lymphoma and the pattern of lymphoma dissemination and growth, suggesting the important value of LTB in improving follicular structure and restoring normal immune environment in lymphoma(Pepe et al. 2018). Although the above viewpoints are preliminary conclusions based on existing research findings, the role and potential applications of LTB may vary in different tumor types and individuals.

This study has some limitations. Firstly, our data is retrospective, and prospective cohort studies are needed to validate the model and increase the reliability and generalizability of the results. Secondly, the model is solely based on gene expression data from skin tissues. To better suit clinical applications, it is recommended to develop biomarkers based on urine or blood samples. These non-invasive sample collection methods are convenient and can be widely implemented. Lastly, future research should include in vivo and in vitro experiments to further validate the mechanisms of action of the cytokines in the study. Through these experiments, we can gain a more comprehensive understanding of the specific functions and interactions of each molecule in the cellular environment. Overcoming these limitations and continuously exploring will improve the credibility and effectiveness of the research in clinical applications.

## Conclusion

Generally speaking, we attempted to develop a new scoring system through the analysis of public database data, aiming to assess the risks associated with disulfidptosis and predict the prognosis of CM patients, thus influencing the selection of immunotherapy. This provides clinicians with a reliable basis for decision-making, enabling them to make personalized treatment choices. Additionally, we conducted in vitro experiments to validate HLA-DQA1 and found it to be a tumor-suppressing gene. Therefore, HLA-DQA1 may be considered a promising target for the treatment of CM patients.

### Supplementary Information

**Additional file 1. Fig. S1A**. The distributions of OS status and risk score in the training cohort. **B**. The PCA and t-SNE plot of the training cohort.

## Data Availability

Data for this study were obtained from public databases.
