# Supplementary figures and images for: Leveraging a disulfidptosis-related signature to predict the prognosis and immunotherapy effectiveness of cutaneous melanoma based on machine learning
Source: Mol Med. 2023 Oct 26;29:145. doi: 10.1186/s10020-023-00739-x (PMC10601311; doi:10.1186/s10020-023-00739-x)

**A**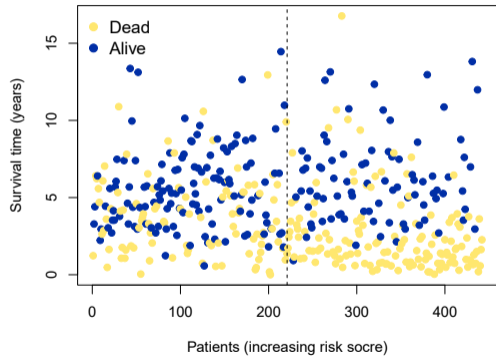**B**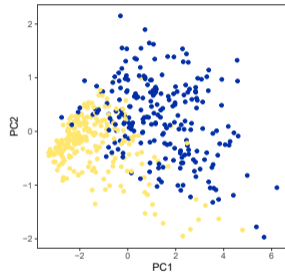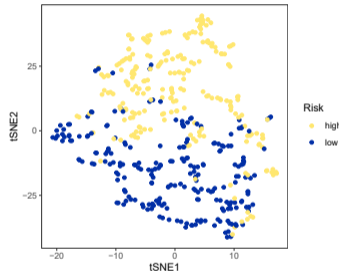

Supplement: Supplementary file 1 — Additional file 1. Fig. S1A. The distributions of OS status and risk score in the training cohort. B. The PCA and t-SNE plot of the training cohort. [file 10020_2023_739_MOESM1_ESM.pdf]
